# Rare complication after thyroidectomy-cervical esophageal stenosis: a case report and literature review

**DOI:** 10.1186/1477-7819-12-308

**Published:** 2014-10-11

**Authors:** Hanwei Peng, Steven J Wang, Weixiong Li

**Affiliations:** Department of Head and Neck Surgery, Cancer Hospital of Shantou University Medical College, 7 Raoping Road, Shantou City, Guangdong Province 515031 China; Department of Otolaryngology-Head and Neck Surgery, University of California-San Francisco, 2233 Post St, 3rd Floor, San Francisco, CA 94115 USA; Department of Head and Neck Surgery, Chaozhou People’s Hospital, Chaozhou City, Guangdong Province 521011 China

**Keywords:** Cervical esophageal perforation, Cervical esophageal stenosis, Reconstructive surgery, Thyroid carcinoma, Thyroidectomy, Tubed forearm free flap

## Abstract

The most common complications after thyroidectomy are injuries associated with the recurrent laryngeal nerve and parathyroid gland. Cervical esophagus perforation is an exceptionally rare complication after thyroidectomy; it can usually be resolved by conservative care. Cervical esophageal stenosis secondary to intraoperative esophageal injury during thyroidectomy is much rarer and has not been reported in the literature to date. We report a case of esophageal stenosis following thyroidectomy performed at a peripheral hospital. The patient initially underwent a thyroidectomy for papillary thyroid carcinoma involving the cervical esophagus; esophageal perforation was noted intraoperatively, and closed using three number 4 silk sutures. Cervical esophageal stenosis subsequently developed after conservative care. The patient was successfully treated with cervical esophagectomy and reconstruction using a tubed forearm free flap after a failed attempt at endoscopic recanalization. This case is discussed in conjunction with a review of the literature.

## Background

The etiology of esophageal stricture includes peptic stricture, pills, neoplasms, infections, irradiation, sclerotherapy, caustic substances, systemic diseases, extrinsic compression, and congenital esophageal stenosis [1]. Iatrogenic esophageal strictures frequently result from treatment (for example, esophagogastric anastomosis, radiation, or chemoradiation) of esophageal carcinoma or congenital esophageal atresia [2]. Maltreated esophageal perforations can sometimes develop into esophageal stricture. To our knowledge, there is no report on iatrogenic cervical esophageal stricture resulting from thyroidectomy in the literature.

Studies of post-thyroidectomy complications from large patient cohorts demonstrate that common complications associated with thyroidectomy include recurrent laryngeal nerve palsy, superior laryngeal nerve injury, hypoparathyroidism, hemorrhage, and wound infection; whereas, thoracic duct injury and ulnar nerve injury due to malpositioning on the operating table were reported as rare complications [3, 4]. Tracheal or esophageal wounds resulting from thyroidectomy are not reported in the literature except as isolated case reports [5–10]. Although rare, cervical esophageal perforation does occur in practice, and can usually be safely resolved with intraoperative closure of the esophageal mucosa or postoperative drainage. However, if the esophageal perforation is not recognized or managed appropriately, a more refractory complication can occur, for which radical surgical intervention may be necessary.

We report a case of cervical esophageal stenosis that resulted from thyroidectomy performed at a peripheral hospital; the stenosis was successfully treated by cervical esophagectomy with larynx preservation and reconstruction with a tubed radial forearm free flap. We also discuss the prevention and treatment options for this complication with a review of the literature.

## Case presentation

**Figure 1:**
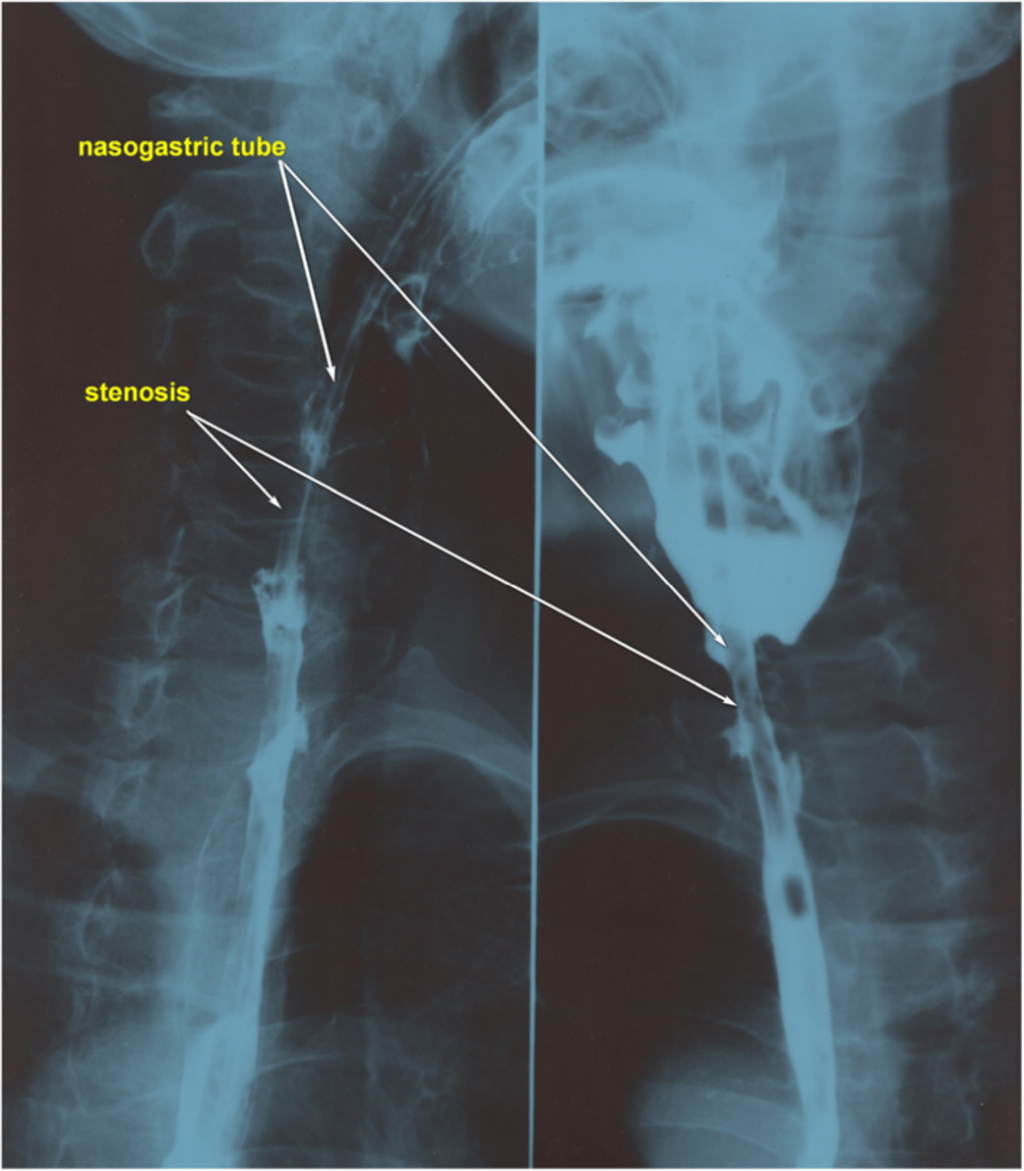
**Esophageal barium radiography on the 7th postoperative day revealed severe stenosis of the cervical esophagus.**

An otherwise healthy 66-year-old woman presented with a 4 cm diameter mass in the left thyroid lobe, which she had had for 2 months, and underwent a thyroidectomy at a peripheral hospital in September 2011. No preoperative imaging study was performed other than a color ultrasonography. Intraoperative frozen section revealed a papillary thyroid carcinoma, and the patient underwent a thyroid lobectomy without lymph node dissection. Intraoperative exploration revealed that the musculature of the cervical esophagus was involved with tumor. Elaborate efforts were made to remove the tumor en bloc; cervical esophageal perforation was identified intraoperatively and closed primarily with three number 4 silk sutures. The patient developed hoarseness, aspiration, swelling on the left side, discharge from the incision line, and high fever on the first postoperative day. Oral intake was withheld; intravenous nutrition and antibiotics were administered. Esophageal barium X-radiography on the seventh postoperative day revealed severe stenosis of the cervical esophagus 2 cm below the inlet (Figure 1), which was subsequently verified by a transnasal gastroscope. Although the endoscope could not pass the stenosis, a nasogastric tube was successfully inserted for enteral nutrition. Wound drainage was maintained for 2 weeks. Daily drainage of a mixture of abscess and saliva was 80 to 150 ml from the 1st to the 7th postoperative day. The drainage diminished slowly and the wound healed; the drain was removed on the 14th postoperative day. On the 10th postoperative day, the patient began to complain of dysphagia and could not swallow any saliva. The nasogastric tube was removed and gastrostomy was performed for long-term nutrition 2 weeks later. An interhospital consultation was obtained for further diagnosis and management 2 months after the operation, when complete dysphagia persisted. Computed tomography demonstrated a stricture of the cervical esophagus with a length of 3 cm just 2 cm below the inlet, without residual tumor or lymph node metastasis. Fiberscopy revealed a complete stenosis of the esophagus. Fiberscopic dilation was attempted but failed because the smallest fiberscope could not pass the esophageal lesion.

**Figure 2:**
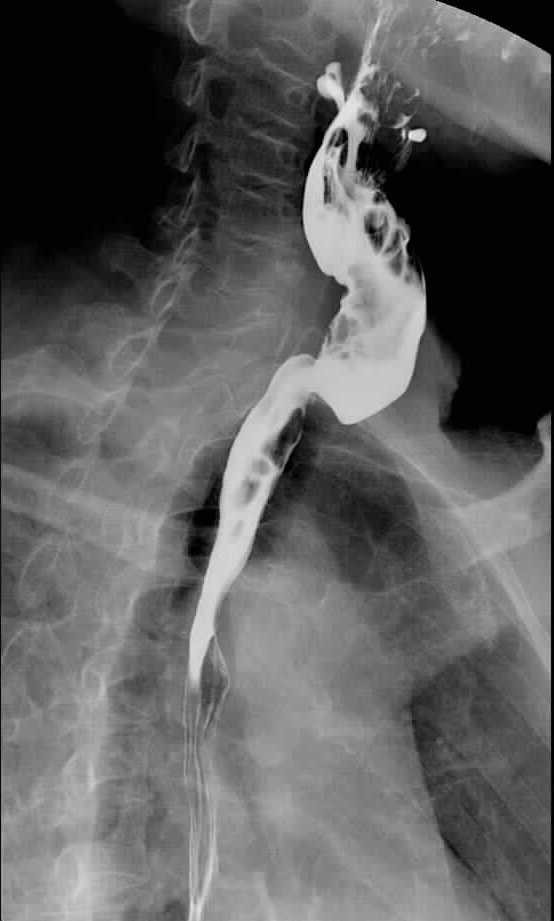
**Barium radiography 6 months after the operation demonstrated that the reconstructed cervical esophagus was widely patent and smooth.**

After informed consent was obtained from the patient and her family, the patient was taken to surgery. Intraoperative exploration found that the anterior wall of the cervical esophagus was sutured to its posterior wall and that the esophagus was completely closed by granulation and fibrotic tissue 2 cm below the inlet; the recurrent laryngeal nerve had been transected during the initial surgery. A cervical esophagectomy with preservation of the larynx was performed that resulted in a circumferential defect of 6 cm length, starting at the esophageal inlet. A tubed radial forearm free flap of 12 × 7 cm^2^ was used to reconstruct the circumferential esophageal defect. The donor artery was anastomosed end-to-end to the transcervical artery and the accompanying vein was anastomosed end-to-side to the internal jugular vein. Postoperative recovery was uneventful. The patient was given a trial of water oral intake on the 7th postoperative day without any discomfort and subsequently began a liquid diet. One month later, she tolerated a normal diet. Subsequently, the patient underwent radioactive iodine ablation. Barium radiography 6 months after the operation demonstrated that the reconstructed cervical esophagus was widely patent and smooth, with a diameter of 2.5 cm (Figure 2).

## Discussion

Iatrogenic cervical esophageal perforation occurring during thyroidectomy by an experienced thyroid surgeon is considered rare, mostly appearing in the literature as case reports [7, 11]. However, when a thyroidectomy is performed by a surgeon not specialized in thyroid surgery, which is common in peripheral hospitals in China, the incidence of esophageal injury may be higher. In the USA, higher complication rates have been observed among low-volume thyroid surgeons than among experienced thyroid surgeons in high-volume centers [12].

The incidence of upper aerodigestive tract invasion by well-differentiated thyroid carcinoma ranges from 1% to 16%; esophageal invasion is found in approximately one-fifth of these patients [13, 14]. However, it has been reported that the invasion is usually confined to the muscular layer and spares the mucosa and submucosa [13]. Papillary carcinoma of the thyroid is the most common histopathologic variant that invades the upper aerodigestive tract; medullary carcinoma rarely does so. Although anaplastic carcinoma may also invade these structures, because this disease is almost invariably fatal, surgery in such patients is chiefly limited to acute airway management. When a full-thickness esophageal defect is created from tumor exenteration, primary closure can be performed if the closure is not under tension and if the tissue is non-irradiated and healthy [13]. An unidentified cervical esophageal perforation rarely occurs under experienced hands [3]. In this reported case, a penetrative defect was noticed after resection of the involved cervical esophagus, and primary closure was attempted with three number 4 silk sutures. Unfortunately, a postoperative esophageal fistula developed. We believe that this was probably due to inadequate exposure of the esophageal lumen and improper suture repair technique. As a result, the sutures penetrated the anterior wall of the cervical esophagus 2 cm inferior to the inlet, as verified by intraoperative exploration during the salvage surgery.

When a cervical esophageal perforation is identified postoperatively, potential causes should be analyzed, to guide further management. If the perforation is the result of a simple mucosal leak, a wound drain should be placed, oral intake withheld, nutrition support (intravenous or enteral) instituted, and antibiotics administered. For this case, endoscopic examination was not done until the 7th postoperative day, eliminating the possibility of early surgical intervention that might have led to a better outcome.

Endoscopic guided dilation with bougies, balloons, or stents has been reported to be an effective technique for cervical esophageal stenosis, particularly if from benign etiology [2, 15–17]. However, endoscopic dilation is not possible when a fiberscope cannot pass the stricture site. In this case, the esophageal lumen was completely blocked, owing to granulation and fibrosis. An esophagectomy with reconstruction of the digestive tract was believed to be the last resort for this patient. Gastric pull-up, jejunum flap, and other free cutaneous or myocutaneous flaps have been reported as successful reconstructive methods for circumferential cervical esophagus defects [18, 19]. With regard to our case, we chose a radial forearm free flap to reconstruct the defect resulting from the resection of the atretic cervical esophagus with preservation of the larynx, considering the flexibility, thinness, and reliability of this flap. However, a gastric pull-up was not an ideal method because the patient had undergone a previous gastrostomy that might increase the risk of surgical complication, and a gastric pull-up would also require an abdominal laparotomy, with its potential morbidity. Similarly, a jejunum flap, although having the advantage of being a mucosal epithelial-lined tubed lumen, was not considered to be the best choice because of the greater potential morbidity of the donor site harvest. Other cutaneous or myocutaneous flaps (for example, an anterolateral thigh flap or a pectoralis major flap) would have been too bulky for the reconstruction and might have led to cosmetic problems or swallowing dysfunction. The patient and her family were satisfied with the functional and cosmetic outcome of the tubed forearm flap reconstruction. The follow-up barium radiography demonstrated that the reconstructed cervical esophagus was smooth and widely patent, with a diameter of 2.5 cm.

## Conclusions

Esophagus stenosis resulting from thyroidectomy is a very rare complication that can be avoided by adequate preoperative evaluation, appropriate intraoperative management, early diagnosis, and early surgical intervention. When conservative endoscopic dilation fails, a larynx preserving cervical esophagectomy with reconstruction utilizing a radial forearm free flap is an effective method that should be considered.

## Consent

Written informed consent was obtained from the patient enrolled in the report. The study protocol conformed to the ethical guidelines of the 1975 Declaration of Helsinki and the guidelines of the regional ethical committees of Cancer Hospital of Shantou University Medical College, China.
